# Assessment of the biological quality of port areas: A case study on the three harbours of La Rochelle: The marina, the fishing harbour and the seaport

**DOI:** 10.1371/journal.pone.0198255

**Published:** 2018-06-25

**Authors:** Marine Breitwieser, Emmanuel Dubillot, Marine Barbarin, Carine Churlaud, Valérie Huet, Frédéric Muttin, Hélène Thomas

**Affiliations:** 1 Littoral Environnement et Sociétés (LIENSs), UMR 7266, CNRS-Université de La Rochelle, La Rochelle, France; 2 EIGSI, La Rochelle, France; Nanjing University, CHINA

## Abstract

This work was designed to investigate biological impacts at 3 dates (day 0, day 7 and day 21) on black scallops (*Mimachlamys varia*) in the three ports areas of La Rochelle town in winter 2017. In order to assess the biological effects on the wild population of black scallops, bivalves were place in four different locations: in the three ports (semi-closed areas), and in a marshland uncontaminated site (closed area). Biomarkers of effects (heavy metals) and exposure (oxidative stress and immunological effects) were assessed in the digestive glands of specimens in order to compare two techniques of sampling: “pool” technique and “inter-subject” technique. Our findings reported in the both techniques show significant modulation of GST (detoxification), SOD (antioxidant response) and MDA (lipid peroxidation) in bivalves exposed to a specific contamination in each port. Laccase-type enzyme also highlighted an important aspect in terms of biomarker response of the immune function at the 7^th^ day of exposition. Overall, our study demonstrated that the “pool” technique using the same quality indicator *M*. *varia* could be used to obtain reliable results at lower costs. In contrast, in fundamental context, the “inter-subject” technique could bring more precise results to light. However, it requires burdensome and costly handling.

## Introduction

From analyses and recommendations given by (European, national and regional) political bodies and research organisations, strong expectations were clearly identified for this case study on port areas including the marina, the fishing harbour and the seaport.

After an expansion in 2014, the marina of La Rochelle is currently the biggest marina in Western Europe (5,000 spaces). The fishing harbour located in La Rochelle is the 4^th^ most important fishing harbour in France. The Atlantic Port of La Rochelle is the 6^th^ largest seaport in France and deals with more than 85% of goods from maritime transport of the Nouvelle-Aquitaine region. The environmental policy of the marina, the fishing harbour and the seaport (Grand Port Maritime) results in actions on land and sea. Indeed, the mostly voluntary involvement in the marina and the Atlantic Port of La Rochelle led to an ISO 14001 certification, which is an international reference in terms of environmental management. These harbours gradually served as references in sustainable development through the implementation of actions, partnerships and incentive policies for port users to respect nature. Despite this outstanding ecological awareness, port operations still have a major impact on the water quality and the marine biodiversity. Marine pollution may have organic, inorganic and biological causes. The equipment and the maritime activity may be source of pollution. Indeed, the boats components and their maintenance (antifouling, sacrificial anodes) are elements of pollution. In addition, runoff and leaching of soils, sources of pollution, lead to contaminations by heavy metals, hydrocarbons and plant protection products. These pollutants are accidentally released in aquatic ecosystems. These various rejections are a real environmental struggle for the fauna and flora of semi-opened environments, in particular for bivalve species such as the black scallop *Mimachlamys varia* [1–3], with significant socio-economic interest (consumed species). To realise this study, digestive gland organ was used as a tool because it has a detoxication role.

The purpose of this study was to (1) assess the impact of inorganic chemical contamination on the state of health of bivalves *M*. *varia* considered by the scientific community as sentinel species [4] and representative of the environmental quality of port area [5] as well as to (2) compare responses at “inter-subject” and “pool” levels (explained page 3) for each site and day of sampling, in order to reduce the cost and time of analysing.

This caging experiment work is innovative because it performed reveals data that have never been described before in these three port areas (marina, fishing and commercial) of the same city.

## Material and methods

Monitoring study with the marine bivalve *M*. *varia* was authorized by the French Ministère de l’Ecologie, du Développement Durable et de l’Energie (Direction interrégionale de la mer Sud-Atlantique) and each harbour private administration. Our field study did not involve an endangered or protected species.

### Monitoring sites and sampling strategy

In spring 2017, a batch of variegated intertidal scallop (*Mimachlamys varia*) from the Tinduff hatchery (Brittany Region), aged of one year and a half with a size of 3.24 ± 0.46 cm of length and 3 ± 0 cm of width was caged in the marsh area for fifteen days in order to acclimate. Then, three groups of specimens are transplanted in the three intra-harbour sites and the marsh. This study was carried out in “caging” (Australian oyster cages) *in situ* in April 2017 at three sampling dates for 21 days (day 0, day 7, day 21). At each experiment day (day 0, day 7, day 21), surviving species in the caging were counted and compared to the initial batch number. Moreover, it was noted a low mortality rate (10%) is recorded after transplantation in the harbour sites.

**Fig 1:**
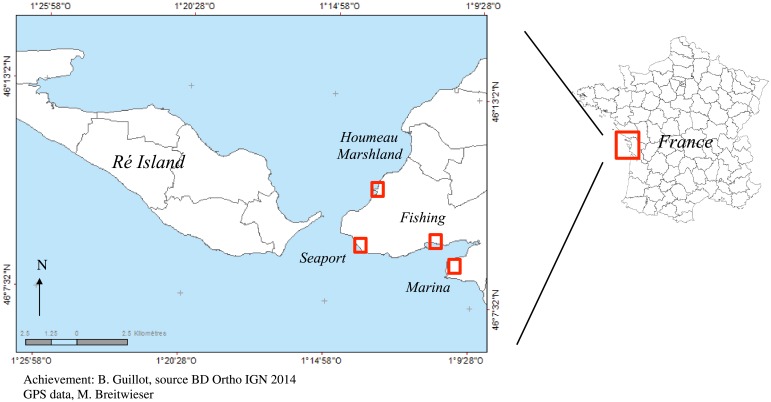
Map of France with the locations of the study areas. Four sampling sites are presented: Houmeau Marshland which is the less-contaminated area with the three habours (Marina, Fishing Harbour and Seaport).

The purpose of this study was to evaluate temporal effects of inorganic contaminants on the state of health of black scallops *M*. *varia*. Fig 1 shows the samples collected at three intra-harbour sites (close to the outlets of rainwater for all harbours) and at the Houmeau oyster marshland, considered as the reference site (closed environment). Variegated scallops were collected from four sampling sites in three habour sites (Marina: 46°14N, -1°16W, Fishing: 46°16N, -1°23W, Seaport: 46°16N, -1°23W, and at the Houmeau Marshland (Marshland: 46°18N, -1°19W).

**Fig 2:**
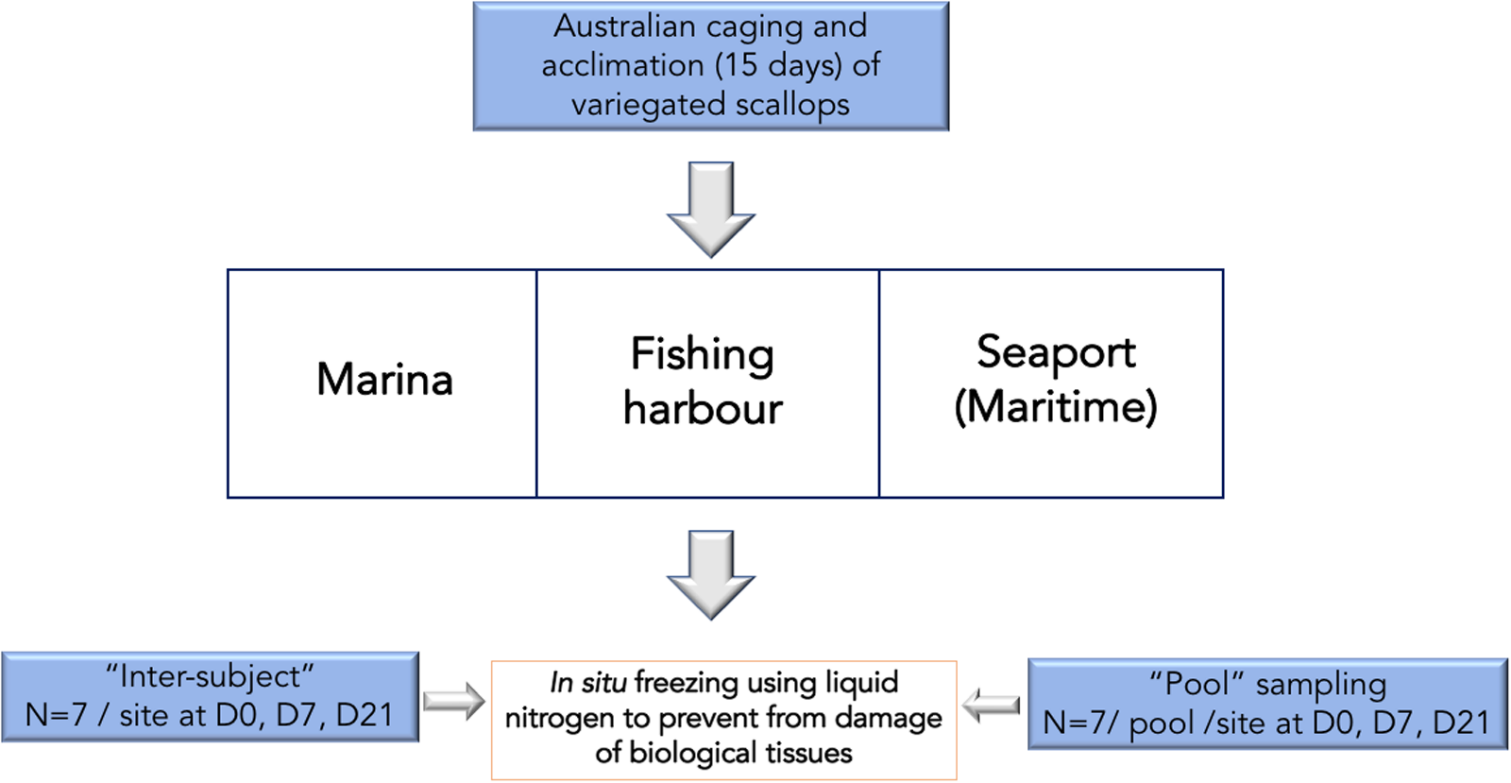
Integrative approach of sampling strategies of bivalves *M*. *varia* at all sampling sites in April 2017.

For each sampling, “pooled” or “non-pooled” species were frozen in liquid nitrogen *in situ* to prevent from enzymatic degradation and damage of biological tissues (Fig 2).

The “pool” technique consists in carrying analyses on a group of subjects (n = 7) ground together (macro-subjects). Conversely, the “inter-subject” technique aims at evaluating individually each of the 7 samplings. In general, this last method requires dealing with a scatter plot through statistical analyses to draw tendencies and correlations.

### Implementation of state of health analysis on bivalve species *M*. *varia*

Four biomarkers of exposure were measured using a spectrophotometer (SAFAS Flx-Xenius) to study the capacities of detoxification (GST), immunity (Laccase), oxidative stress response (SOD), and lipid peroxidation (metabolite MDA). On the same subjects, 14 metals (As, Cd, Co, Cr, Cu, Mn, Fe, Ni, Pb, Se, V, Zn, Ag, Sn) were measured in the same organs (digestive glands) using equipment of the Metal Analyses platform (plateforme Analyses Élémentaires) (ICP-MS, ICP-AES) from the LIENSs laboratory.

### Biomarkers of effects: Heavy metals assay

Trace elements analysis was realized according previous works [4,6]. Analyses of 14 trace elements (Ag, As, Cd, Co, Cr, Cu, Fe, Mn, Ni, Pb, Se, Sn, V, Zn) fractions were digested using a nitric—hydrochloric acid mixture with 1.5mL of 65% HNO3 (FisherScientific, Trace Metal Grade quality) and 0.5mL of 37% HCl (FisherScientific, Trace Metal Grade quality). Acidic digestion was performed overnight under a basket at room temperature and then samples were heated in a microwave oven for 30 min, increasing the temperature to 120 °C, and then for 15 min at 120 °C. After the digestion process, each sample was diluted to 12.5 mL with ultra-pure quality water. Ag, As, Co, Cr, Ni, Pb, Se and Sn were analysed by ICP-MS (ThermofisherElectron^®^ X series II) and Cd, Cu, Fe, Mn, V and Zn were analysed by ICP-OES (Varian^®^ Vista-Pro). Reference tissues (dogfish liver DOLT-4, NRCC, and lobster hepatopancreas TORT-3, NRCC) were treated and analysed in the same way as the samples. The results were in good agreement with the certified values. The recoveries for DOLT4 were: As (104%), Cd (98%), Cu (99%), Fe (100%), Ni (99%), Pb (105%), Se (97%), Zn (101%), Ag (101%) and for TORT-3 were: As (100%), Cd (93%), Co (97%), Cr (99%), Cu (100%), Fe (93%), Mn (95%), Ni (98%), Pb (98%), Se (96%), Zn (100%). All concentrations are expressed on a dry weight basis (μg g−1 dry wt) (Fig 2). All chemical analyses were performed in triplicate.

### Biomarkers of exposure assays

All the biochemical assays were performed with the same instrument: SAFAS Flx-xenius at the specific absorbance for each biomarker kind. Before statistical analysis, row data for GST, SOD and Laccase were expressed in U/mg of proteins. MDA row data concentrations were expressed in μM/mg of protein. Total protein concentrations were determined following the BCA Kit methodology (Bicinchononique Acid Kit, Sigma Aldrich). The BCA kit contained bovine serum albumin (BSA) as a standard and involved the reduction of alkaline Cu^2 +^ by proteins [7] at absorbance 562 nm.

#### Glutathion S-transferase (GST) assay

Glutathion S-transferase (GST) specific activity was measured given its important role in xenobiotic detoxication. Total activity of GST (GST are a group of enzymes) was determined according to the kit method of Sigma (CS0410-1KT).

#### Superoxyde Dismutase (SOD) assay

Superoxide dismutase (SOD) activity was measured given its important role in antioxidant response [8].

#### Molondialdeyde (MDA) assay

Oxidative stress was assessed through lipid peroxidation by quantifying malondialdehyde (MDA) that is a chemical metabolite of cell lipid degradation. MDA concentration was estimated using a commercially available MDA assay kit (Oxis International).

#### Laccase assay

Immune system alteration was evaluated by assessing a phenoloxidase (PO) activity (laccase-type) since this enzymatic activity has been shown to be contaminant sensitive [9] and to play an important role in the immune defense mechanism in invertebrates.

### Data analysis

The data analysis was performed in collaboration with the Ecole d’ingénieurs généralistes EIGSI. Raw data were processed in order to compare the sampling dates, the biomarkers of effect (heavy metals) and exposure (SOD, Laccase, MDA and GST), and the sites.

**Fig 3:**
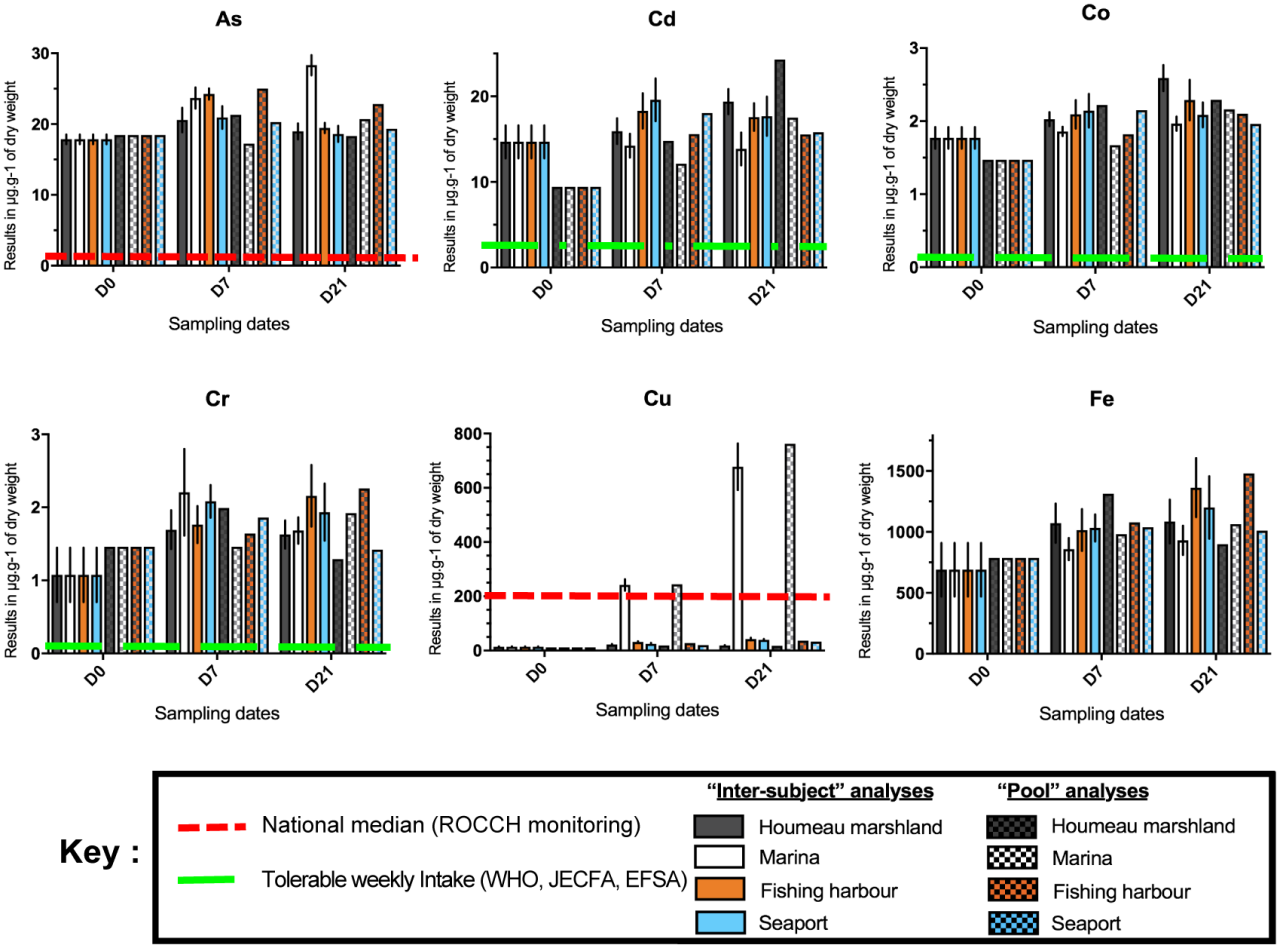
Heavy metals (As, Cd, Co, Cr, Cu, Fe) in the digestive glands of black scallops *M*. *varia* assessed for all sites (the marshland, the marina, the fishing harbour and the seaport) in 2017 at three sampling dates (D0, D7 and D21). These graphs indicate both “inter-subject” (n = 7/site/sampling) and “pool” analysis.

**Fig 4:**
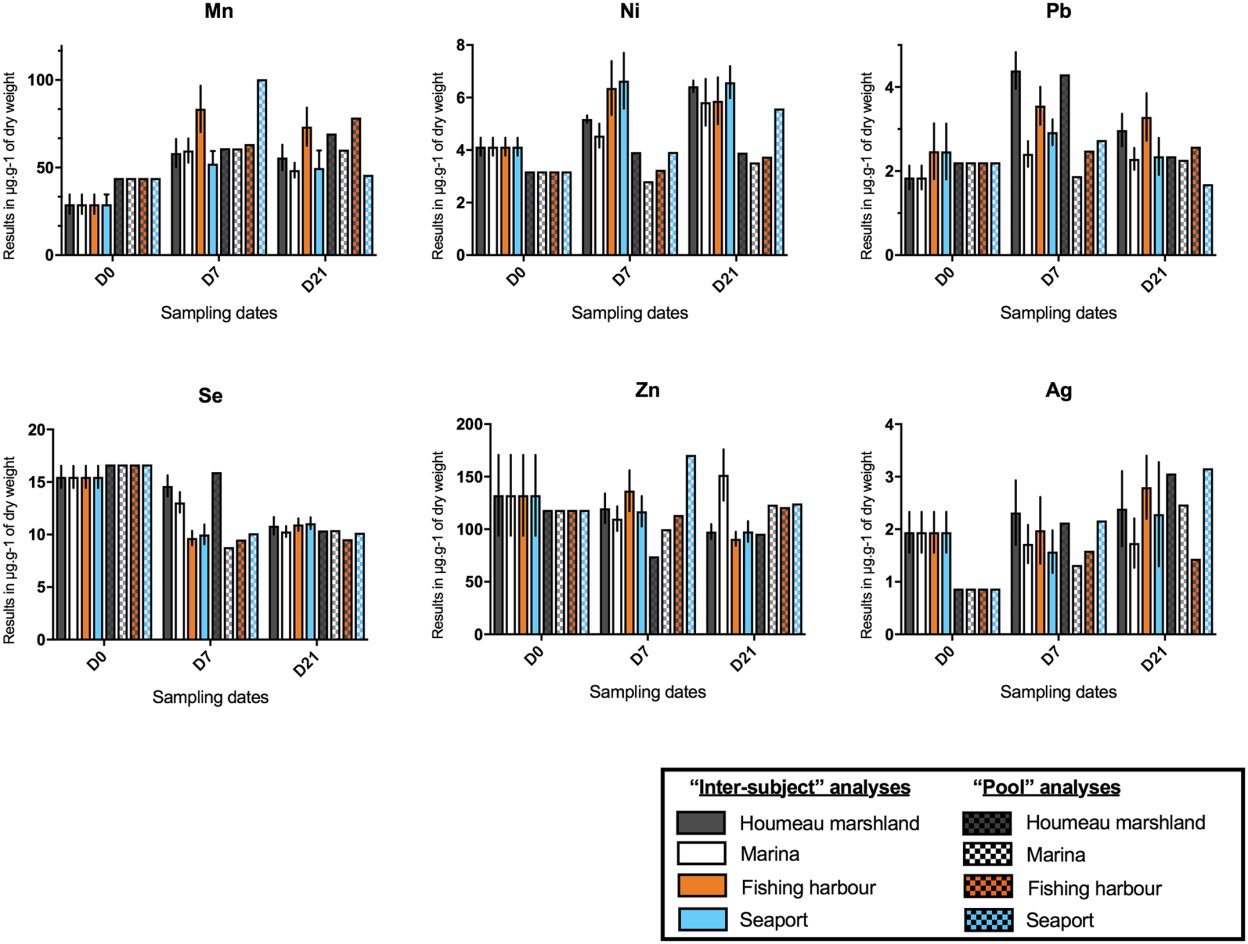
Heavy metals (Mn, Ni, Pb, Se, Zn, Ag) in the digestive glands of black scallops *M*. *varia* assessed for all sites (the marshland, the marina, the fishing harbour and the seaport) in 2017 at three sampling dates (D0, D7 and D21). These graphs indicate both “inter-subject” (n = 7/site/sampling) and “pool” analyses.

**Fig 5:**
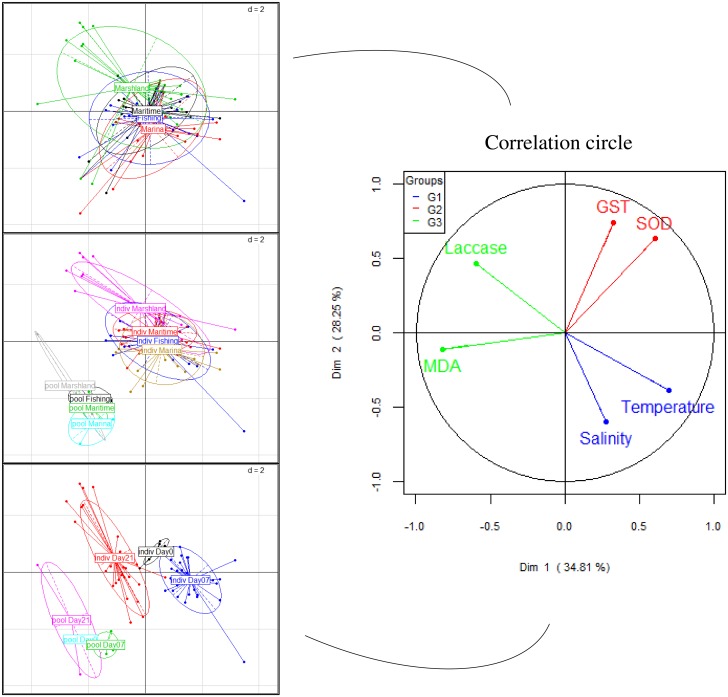
Two-dimensional graph (axes 1 and 2) including the circle of correlations (on the right), the scatter plots comparing each site, the analysis techniques, and the sampling dates (on the left).

**Fig 6:**
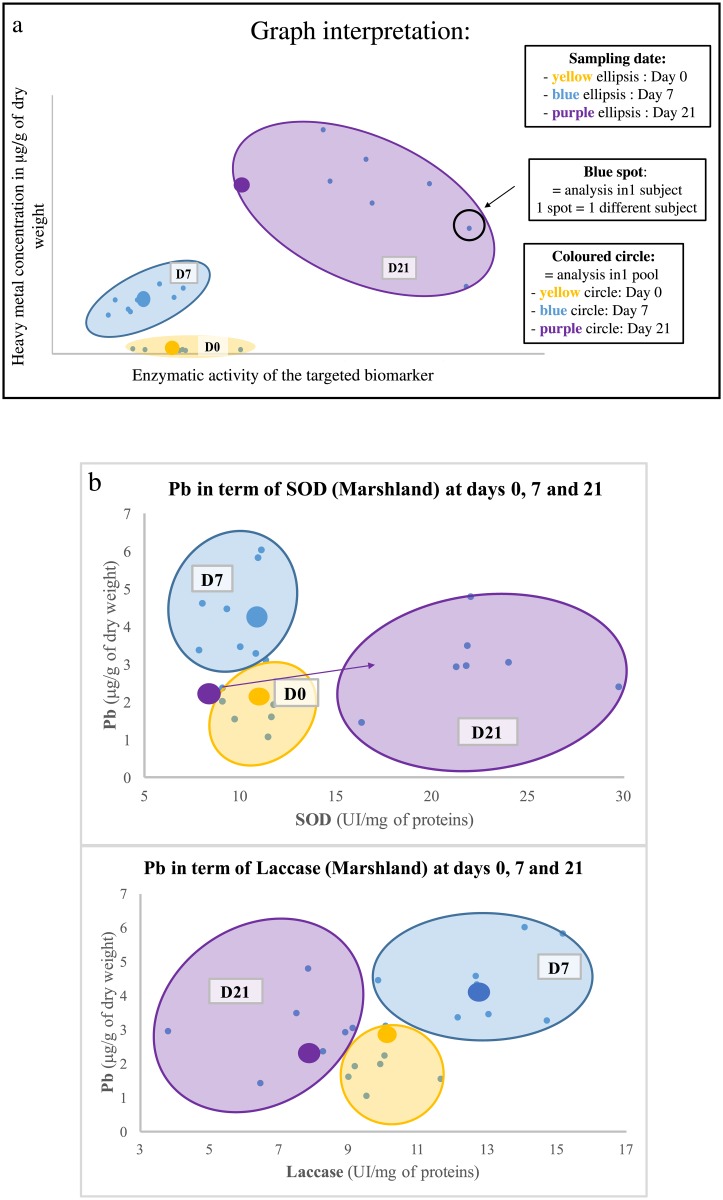
a Graph interpretation illustration for all graphics below, and b Specific pollution of Pb depending on SOD (struggle against ROS) and Laccase (immunomodulation) at the Houmeau marshland site.

**Fig 7:**
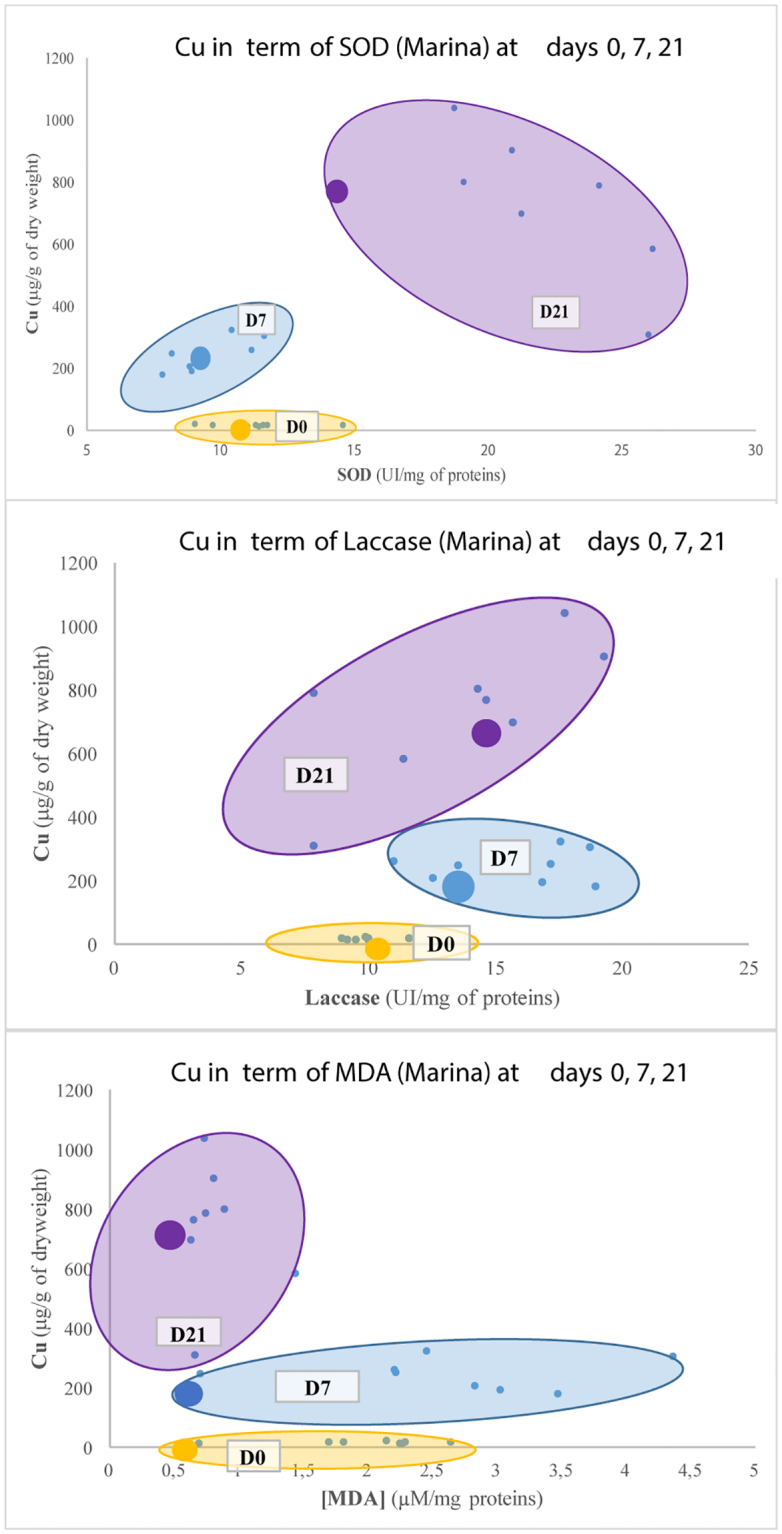
Specific pollution of Cu depending on SOD (struggle against ROS), Laccase (immunomodulation) and MDA (destruction of cell membranes) at the marina of La Rochelle.

**Fig 8:**
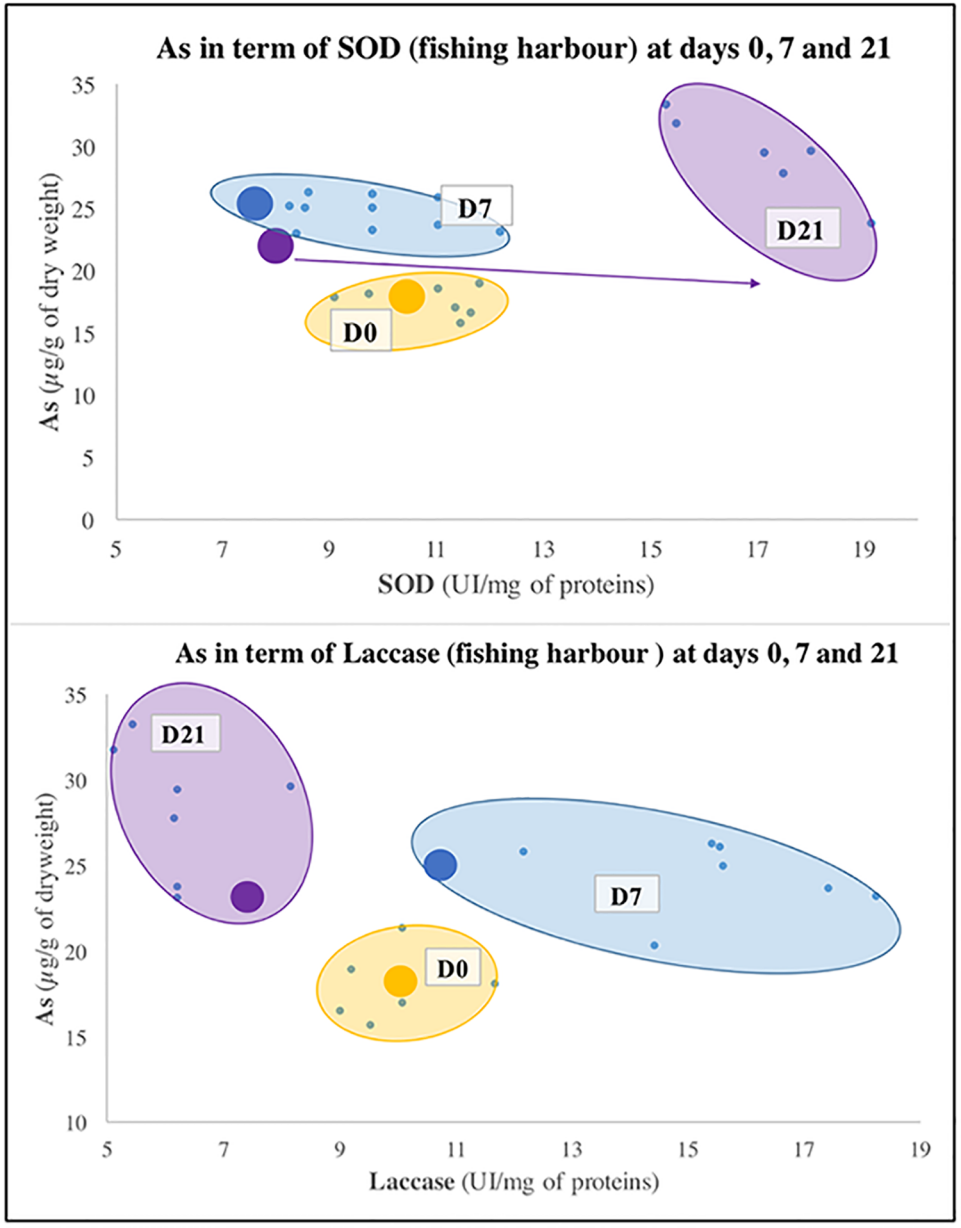
Specific pollution of as depending on SOD (struggle against ROS) and Laccase (immunomodulation) at the fishing harbour.

**Fig 9:**
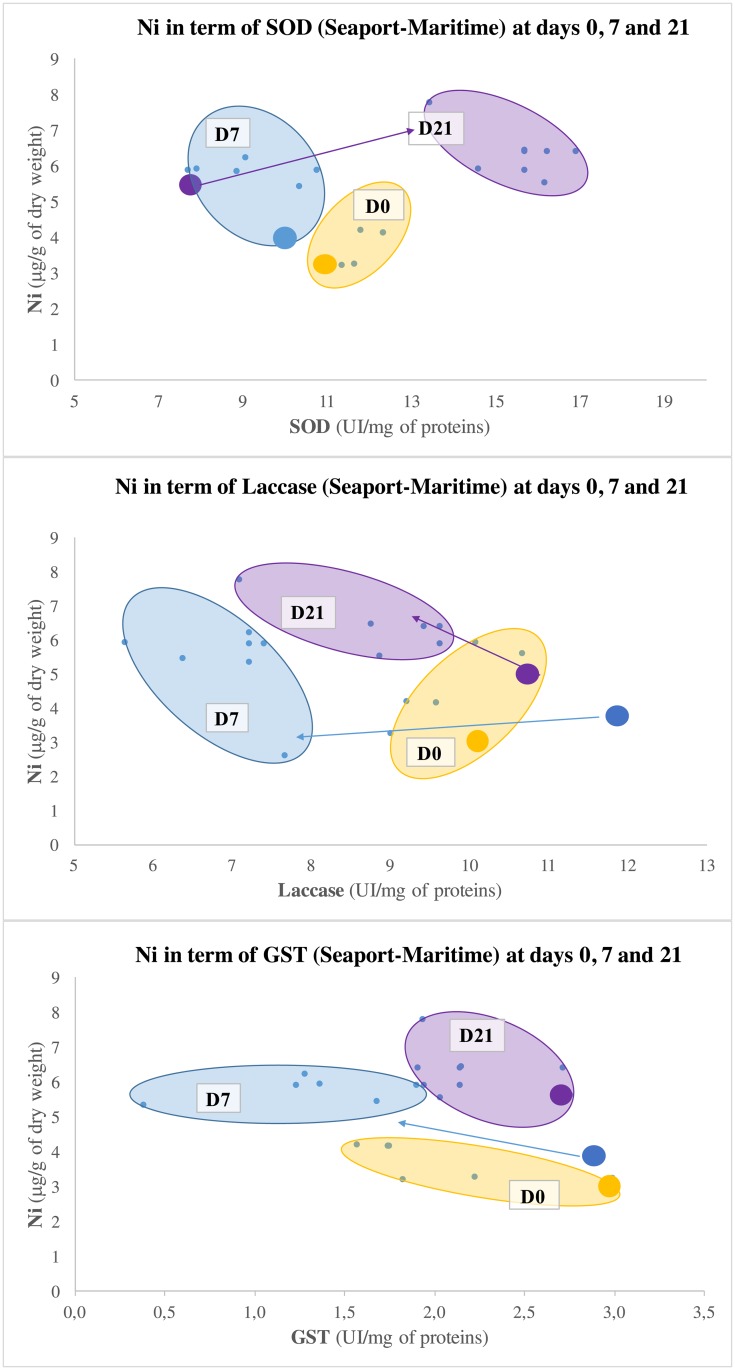
Specific pollution of Ni depending on SOD (struggle against ROS), Laccase (immunomodulation) and MDA (destruction of cell membranes) at the seaport (Maritime).

With this aim in mind, illustrations such as histograms (Figs 3 and 4), Principal Component Analyses (PCA, Fig 5) and graphs of correlations (Figs 6, 7, 8 and 9) were created using Graph Pad Prism (version 7.0c), R version 3.1.2 [10] and Excel 2017. The PCA identifies major linear combinations of variables [11] and extreme values.

Data consist in 4 sites, 3 sampling dates, 16 biomarkers: 4 of exposure (GST, SOD, MDA, Laccase) and 14 of effect (heavy metals). They were analysed following two techniques: the techniques of “inter-subjects” and “pools” of 7 specimens ground together (n = 7). One geographical site is a natural protected area while the three others is located inside three kinds of harbour. The first sampling date corresponds to time zero of the experimentation. These spatio-temporal data are discrete functions. At the contrary, the biomarkers (exposure and effect) are continuous positive functions. The biomarkers of exposure have always strictly positive values. The biomarkers of effect never reach a strictly zero value. When this biomarker measures a heavy metal concentration under a detection threshold, a qualitative neglect ability effect hits the numeric value.

First, biomarkers of exposure were combined to sites and periods (PCA). Then, biomarkers of effect were averaged on sites and periods. Finally, significant biomarkers (of exposure and effect) were associated. The “inter-subject” and “pool” techniques were compared to striking tendencies of biomarkers of effect (Tables 1 and 2).

**Table 1:** Reliability or difference between “inter-subject” (n = 7) and “pool” studies at each site (marshland, marina, fishing harbour and seaport-Maritime) at D21 for the 4 biomarkers of exposure (GST, SOD, MDA and Laccase). Green shading represents 0–20% difference, red shading represents 20–30% difference and yellow shading represents more 30% difference. Negative percentages are lower rates in the “inter-subject” study compared to the “pool” analysis.

	Biomarkers of exposure			
	GST	SOD	MDA	Laccase
**Marshland**	-79%	59%	28%	-8%
**Marina**	-39%	35%	23%	-9%
**Fishing harbour**	-31%	49%	33%	9%
**Seaport-Maritime**	-38%	54%	50%	-27%

**Table 2:** Reliability or difference between “inter-subject” (n = 7) and “pool” studies at each site (marshland, marina, fishing harbour and seaport) at D21 for the 14 heavy metals called biomarkers of effect. Green shading represents 0–20% difference, red shading represents 20–30% difference and yellow shading represents more 30% difference. Negative percentages are lower rates in the “inter-subject” study compared to the “pool” analysis.

	Biomarkers of effect													
	As	Cd	Co	Cr	Cu	Fe	Mn	Ni	Pb	Se	V	Zn	Ag	Sn
**Marshland**	7%	-25%	12%	21%	6%	17%	-10%	35%	21%	4%	52%	2%	14%	25%
**Marina**	-9%	-26%	-10%	-14%	-5%	-14%	-24%	41%	1%	-1%	1%	19%	-42%	-1%
**Fishing harbour**	19%	12%	8%	-5%	14%	-9%	-7%	43%	22%	13%	21%	-33%	49%	10%
**Seaport-Maritime**	1%	11%	6%	27%	18%	16%	8%	13%	28%	8%	34%	-27%	-38%	19%

## Results and discussion of parameters analysed

### Biomarkers of effect: Heavy metals

Figs 3 and 4 present the results of the analyses on heavy metals performed on the digestive glands of the black scallop *M*. *varia* except for tin (Sn) and vanadium (V) with the majority of their values below the quantification limit. These results are presented in μg/g of dry weight.

Regulatory values in μg/g of dry weight are stated for 9 trace elements (references in red and green dotted lines): Ag, As, Cu, Ni, Pb, Zn, Cd, Co, Cr according to the ROCCH monitoring networks (oyster matrix) from IFREMER as well as the Tolerable Weekly Intake (TWI) from the World Health Organization (WHO), the Joint Expert Committee FAO/WHO on Food Additives (JECFA) and the European Food Safety Authority (EFSA). These medians and values are shown for comparison.

By comparing these values with the ones of the monitoring networks IFREMER (ROCCH) and of the TWI and from previous studies in pectinids [2,4,12,13] for 9 heavy metals, results reveal a low contamination by Zn and Ag at all 4 sampling sites, and a low contamination by Cu at the fishing harbour, the Atlantic Port (Seaport-Maritime) and the marshland; an equal contamination by Cu between “pool” and “inter-subject” analyses at D7 at the marina; a higher contamination by As, Cd, Co, Ni and Pb at all sites, and a higher contamination by Cu at Day 21 at the marina.

The intakes on these heavy metal concentrations could result from various by-products such as phosphate fertilisers, the metalworking industry, car combustion from river basin levels and the nature of geochemical backgrounds [14]. In addition, specific pollution at each site was highlighted through linear correlations (Pb at the marshland, Cu at the marina, As at the fishing harbour and Ni at the seaport) and presented under section 3.3. Let us not forget that these points should be made with caution seawater could play a role in general bioaccumulation of inorganic contaminants in bivalves [15]. The capacities of inorganic contaminants in bivalve organisms can differ according tissue characteristics: gills, digestive gland [16]. Here, for the both techniques “pool” and “inter-subject” studies focused on the digestive gland. Indeed, it is important to underline a higher bioaccumulation in the digestive glands of bivalves, mostly for essential metals [5,17–20]. The role of the digestive gland in the trophic pathway may be the main pathway of accumulation for this organ. Moreover, seawater could also play an important role in global bioaccumulation of heavy metals [21].

### Biomarkers of exposure: SOD, GST, MDA, Laccase

The enzymatic assays underlining the state of health of bivalves were performed in order to study the capacities of detoxification of xenobiotic (GST) and to assess the struggle against reactive oxygen species (ROS), the integrity of cell membranes, the involvement of lipid peroxidation (MDA), and the modulations of the immune system (Laccase). Fig 5 displays an easy-to-read, descriptive and multi-criteria analysis in order to compare the results depending on the analysis techniques used (“inter-subject” or “pool”), the sampling dates and the various sites. As shown in the circle of correlations drawn on Fig 5, Laccase and MDA (in green), representing respectively the immunomodulation and the destruction of cell membranes, react independently of the other two biomarkers of exposure (GST and SOD in red). GST and SOD are positively correlated.

The scatter plots displayed on the graphs on the left (Fig 6) indicate: the three types of harbours present mixed results; the marshland is assessed separately from the other three port areas, which are affected by abiotic factors; clear distinction between the “inter-subject” and “pool” analyses; a development of the enzyme activity response from day 21 for the two techniques. Antioxidant systems have been shown to exhibit seasonal variations in relation to temperature, reproductive cycles, and availability of food for different species of molluscs [22,23]. The biomarker activities, which are superior in the marshland, could be due to a higher temperature at day 21 (17°C) in comparison with the three other harbour sites (15°C).

Overall, the results of the specific activities of the four biomarkers assessed by Principal Component Analyses (PCA) and describing the biological effect on the 4 different sites indicate that the state of health is little impacted at all locations. Under normal conditions, the species’ defence systems are able to eradicate permanently and effectively primary free radicals [24,25].

However, in the event of chronic pollution, the organism faces an increase in ROS, which leads to a greater activation of defence enzymes (GST and SOD) and a decrease in the functioning of the immune system [9,26].

The marshland site, which is a reference site, is impacted by the Laccase activity, which seems to be negatively altered. This phenomenon is consistent with the results of previous studies [5]. In addition, the results of higher enzyme activities of the GST and SOD enzymes using the “inter-subject” technique suggest that the black scallops are more sensitive to the first two sampling dates (D0 and D7) as been reported in previous studies [5,27,28]. In contrast, the samples analysed using the “pool” technique reveal a lower MDA concentration than the black scallops assessed with the “inter-subject” method. It seems that the lipid activity and, thus, a mass production of free radicals do not appear when using the “pool” technique [29,30].

### Specific pollution at each site

Statistical and mathematical analyses brought linear correlations to light. These enabled to highlight specific contamination at each site. They are presented in Figs 6, 7, 8 and 9. The specific contaminants are:

- Pb at the marshland;
- Cu at the marina;
- As at the fishing harbour;
- Ni at the seaport (Grand Port Maritime).

Fig 6 demonstrates the connections between the inorganic metal Pb and the biomarkers of effect (SOD and Laccase) at the marshland site. Only the sampling dates D0 and D7 display a homogenous distribution of the spots representing the “inter-subject” and “pool” techniques between Pb and SOD. In contrast, a homogeneous distribution of the Laccase enzymes appears at the three sampling dates between the two methods. A principal component analysis identifies SOD and Lacasse as major variables for representing the marshland site between the fours biomarkers of effect.

Fig 7 presents the interactions between the inorganic metal Cu and the biomarkers of effect (SOD, Laccase and MDA) at the marina. The results show a homogeneous distribution at the three sampling dates between the two methods. The Cu concentration appears as a strictly increasing function in term of time.

Fig 8 illustrates the relationships between the inorganic metal As and the biomarkers of effect (SOD and Laccase) at the fishing harbour site. The results are similar to the ones assessed at the marshland (Michel, 1993). Only the sampling dates D0 and D7 indicate a homogeneous distribution of the spots representing the “inter-subject” and “pool” techniques between As and SOD. However, a homogenous distribution of the Laccase enzymes appears at the three sampling dates between the two techniques. The linear correlation coefficients between As and the four biomarkers of effect show higher values with SOD and Laccase than with GST and MDA. For this reason, SOD and Laccase have been chosen as predictor variables for As concentration dependant variable.

Fig 9 presents the connections between the inorganic metal Ni and the biomarkers of effect (SOD, Laccase and GST) at the seaport (Grand Port Maritime). The analyses for this site reveal a less homogeneous distribution between the “inter-subject” and the “pool” techniques than the other three sites. Only the graphs representing SOD and GST using the “inter-subject” technique follow a similar pattern for the seaport and the three other sites. It was noted that the small level of Ni concentrations measured appearing frequently lower than the detection threshold. Consequently, only a reduce number of subjects has been considered here compared to the others inorganic metals presented before.

As mentioned in section 3.1, the specific nature of lead, copper, arsenic and nickel pollution could result from, respectively, vehicle fuel, antifouling paints, metal structural adhesives and wood protection agents. In the short run, these heavy metal concentrations could lead to a SOD enzyme activity involved in the struggle against ROS [4,31]. In addition, considering their physiology (filter feeders and sedentary species) and their ability to bioaccumulate, the state of heath of scallops can be altered as noticed at all sites at the last sampling date [32,33]. In general, this modulation is characteristic of an increase in the detoxification of xenobiotic-type elements and a decrease in the immune system (Laccase).

### Differences between “inter-subject” and “pool” studies

The purpose of this study is to decide on which technique to use when assessing the state of health of filter feeders organisms. Therefore, reliability analyses for each biomarker were carried out at day 21. These results are presented in Tables 1 and 2.

At day 21, Table 1 displays significant differences in results for three biomarkers of exposure (GST, SOD and MDA) between the two techniques. In contrast, the biomarker representing the immune system modulation (Laccase) shows little difference (less than 20%). The differences in term of geographical site are always negative for GST and positive for SOD and MDA. A negative difference occurs when the “pool” value in a site is higher than the “inter-subject” one.

The differences between techniques in the Table 1 show that 62.5% of total values displayed in the table differ more than 30%. Thus, the “pool” technique appears reliable particularly for the MDA and Laccase, already for marshland and marina areas, contrary to GST and SOD biomarkers.

Table 2 demonstrates the difference between the “inter-subject” and the “pool” techniques at day 21. 65% of total values displayed in the table differ from less than 20%. The “pool” technique appears reliable particularly for the As, Co, Cu, Fe and Se heavy metals in any of the four sites.

To conclude this part, this new approach, used in three harbours located in the same city, enabled to showcase the connection between bioaccumulation (contamination), the state of health and mainly, the response difference between the technics of bivalves *M*. *varia* to assess the biological quality of environments.

## Conclusion

This study enables to understand the advantages and disadvantages of the two methods. To deal with the environmental issues impacting on the various harbours in La Rochelle, the “pool” technique using the same quality indicator *M*. *varia* could be used to obtain reliable results at lower costs. In contrast, in fundamental context, the “inter-subject” technique could bring more precise results to light. However, it requires burdensome and costly handling.

The advantages using the technique of 7 “pooled” specimens are listed below: *reduction in the inter*-subject variability; lower costs; quickness; less statistical analyses of data.

The disadvantages using the technique of 7 “pooled” specimens are listed below: burden of dissections and preparation of homogenates in the laboratory; less reliable correlations with contaminants as we deal with less values; complexity in assessing the impact of a mix of contaminants on the state of health; impossibility to detect extreme or abnormal values in comparison to “inter-subject” studies.

The technique to use should be defined following specifications determined to target the different needs.
